# Insights into Stoichiometry Adjustments Governing the Performance of Flexible Foamed Polyurethane/Ground Tire Rubber Composites

**DOI:** 10.3390/polym14183838

**Published:** 2022-09-14

**Authors:** Adam Olszewski, Paulina Kosmela, Wiktoria Żukowska, Paweł Wojtasz, Mariusz Szczepański, Mateusz Barczewski, Łukasz Zedler, Krzysztof Formela, Aleksander Hejna

**Affiliations:** 1 Department of Polymer Technology, Gdańsk University of Technology, Narutowicza 11/12, 80-233 Gdańsk, Poland; 2 Institute of Materials Technology, Poznan University of Technology, Piotrowo 3, 61-138 Poznan, Poland; 3 Department of Molecular Biotechnology and Microbiology, Gdańsk University of Technology, Narutowicza 11/12, 80-233 Gdańsk, Poland

**Keywords:** flexible polyurethane foams, ground tire rubber, waste, filler modification, interfacial interactions

## Abstract

Polyurethanes (PU) are widely applied in the industry due to their tunable performance adjusted by changes in the isocyanate index—stoichiometric balance between isocyanate and hydroxyl groups. This balance is affected by the incorporation of modifiers of fillers into the PU matrix and is especially crucial for PU foams due to the additional role of isocyanates—foaming of the material. Despite the awareness of the issue underlined in research works, the contribution of additives into formulations is often omitted, adversely impacting foams’ performance. Herein, flexible foamed PU/ground tire rubber (GTR) composites containing 12 different types of modified GTR particles differing by hydroxyl value (L_OH_) (from 45.05 to 88.49 mg KOH/g) were prepared. The impact of GTR functionalities on the mechanical, thermomechanical, and thermal performance of composites prepared with and without considering the L_OH_ of fillers was assessed. Formulation adjustments induced changes in tensile strength (92–218% of the initial value), elongation at break (78–100%), tensile toughness (100–185%), compressive strength (156–343%), and compressive toughness (166–310%) proportional to the shift of glass transition temperatures (3.4–12.3 °C) caused by the additional isocyanates’ reactions yielding structure stiffening. On the other hand, formulation adjustments reduced composites’ thermal degradation onset due to the inferior thermal stability of hard segments compared to soft segments. Generally, changes in the composites’ performance resulting from formulation adjustments were proportional to the hydroxyl values of GTR, justifying the applied approach.

## 1. Introduction

Polyurethanes (PU) are commonly applied in multiple branches of the industry due to their broad range of easily adjustable properties [1,2]. They are present on the market in the form of foams, elastomers, coatings, adhesives, and others, which enables applications, e.g., in construction, building, automotive, electronic, furniture, or household industries, as seats, cushions, thermal and acoustic insulation materials, sealants, adhesives, coatings, membranes, textiles, varnishes, wheels, and others [3,4,5]. Irrespectively of their type, their performance is driven by their formulations, which are often quite complex and consist of two main components—polyols and isocyanates, but also surfactants, blowing agents, catalysts, chain extenders, and crosslinking agents [6,7]. The detailed type and content of particular compounds significantly impact the structure and performance of polyurethanes [8]. Nevertheless, irrespective of the additional compounds, the most critical is the stoichiometry of the polyaddition reaction between isocyanates and polyols, which generates urethane groups [9]. The ratio of these components, particularly their functional groups, called the isocyanate index, is the main parameter quantitatively describing polyaddition stoichiometry [10]. Its value is most critical for PU foams since the isocyanates, except for the generation of urethanes, are also responsible for the foaming of material in the case of chemical foaming with carbon dioxide generated in the reaction between isocyanates and water [10]. It is a popular approach because it eliminates using physical blowing agents, often harmful to the environment [11].

The isocyanate index can be affected by the presence of electron-donating groups, mostly hydroxyl and amine, due to their high reactivity [12]. Therefore, the presence of moisture in additional compounds containing such functionalities impacts the balance between isocyanate and hydroxyl groups and should be considered when developing PU formulation. The moisture contribution is more accessible to consider due to the high reactivity of isocyanates with water molecules [13]. However, research works often do not consider the impact of other compounds, mainly fillers applied in the manufacturing of PU-based composites. Multiple materials investigated as fillers for PU composites contain nucleophile groups, which can react with isocyanates [14]. As a result, the stoichiometric balance of polyaddition yielding urethane groups is affected along with the structure and performance of resulting composites. The filler-induced disturbance of the isocyanate index affecting the foaming kinetics and cellular structure of PU foams has been reported by Członka et al. [15] and Bryśkiewicz et al. [16]. In order to prepare foamed PU composites deliberately, the contribution of fillers to the isocyanate index should be considered. 

Nevertheless, the impact of filler functionalities is not as straightforward as in the case of moisture. The irregularities are primarily attributed to the complex structure of fillers, either plant-based like cellulose or wood flour or others like leather waste or recycled plastics and rubber [17,18,19,20]. Contrary to the water or polyols particles, the accessibility of hydroxyl groups present in the structure of fillers is significantly lower, primarily due to the steric hindrance [13].

Keeping in mind these issues, it is essential to consider the impact of fillers’ chemical structure during the development of foamed PU-based composites to maximize their efficiency and repeatability. In order to adjust PU formulations properly, the contribution of fillers’ functionalities to the overall isocyanate index should be assessed. In our previous work [21], we proposed the method for the determination of hydroxyl value (L_OH_) of ground tire rubber (GTR) developed by the modification of the method for the determination of free isocyanate group content by titration with dibutylamine, according to ASTM D-2572. The proposed method involves a chemical reaction of GTR with toluene diisocyanate (TDI), so it mirrored the conditions during the preparation of PU/GTR composites. Subsequently, one type of GTR was incorporated into foamed flexible PU matrix, and its contribution to the isocyanate index was considered or not during formulation development [22]. The properties of unfilled PU foams and composites containing deactivated GTR (with blocked hydroxyl groups) were compared. Static tensile and compression tests were performed to evaluate the actual impact of GTR functional groups on the composites’ performance. Tensile-based dependencies indicated that around 23–33% of GTR hydroxyls reacted with isocyanates, while compression tests suggested higher values in the 48–57% range. Therefore, obtained results confirmed the above-mentioned incomplete reactivity of fillers’ functional groups during PU preparation.

The presented research work is an extension of the previous study. It deals with the mechanical, thermomechanical, and thermal performance of flexible foamed PU/GTR composites containing twelve different types of GTR previously subjected to thermomechanical modification in a twin-screw extruder. As presented in previous works [23,24,25,26], such a treatment induces noticeable changes in the chemical structure of GTR, including oxidation reactions leading to the formation of electron-donating groups, which can react with isocyanates. Therefore, the impact of treatment temperature (from 50 to 200 °C) and screw speed (from 50 to 300 rpm) on the GTR hydroxyl value and the performance of resulting PU/GTR composites were assessed. To evaluate the actual impact of GTR functionalities on the prepared composites, two approaches to PU preparation were applied, with and without considering the L_OH_ of GTR fillers in calculations of the isocyanate index.

## 2. Materials and Methods

### 2.1. Materials

During the preparation of flexible foamed PU/GTR composites, Rokopol^®^F3000 and Rokopol^®^V700 produced by the PCC Group (Brzeg Dolny, Poland), as well as glycerol obtained from Sigma Aldrich (Saint Louis, MI, USA), were applied as polyol components. The first two compounds are homogenous, clear, liquid polyether polyols and glycerol-based polyoxyalkylene triols. Rokopol^®^F3000 and Rokopol^®^V700 are characterized by L_OH_ in the range of 53–59 and 225–250 mg KOH/g, respectively. Their molecular weights are 3000 and 700 g/mol, respectively, while dynamic viscosity at 25 °C ranges from 460 to 520 and 220 to 270 mPa·s, respectively. These compounds are characterized by acid values below 0.1 mg KOH/g, water content below 0.1%, and color below 50 Hazen units. The purity of applied glycerol was min. 99.5%, its hydroxyl value equaled 1800 mg KOH/g, while density was 1.26 g/cm^3^. As the isocyanate component was applied SPECFLEX NF 434 purchased from M. B. Market Ltd. (Baniocha, Poland), methylene diphenyl diisocyanate-based component characterized by the brown color, free isocyanate content of 29.5%, dynamic viscosity at 25 °C of 66 mPa·s, and specific gravity at 25 °C of 1.21. Applied PU composition also included three catalysts. The first one was PC CAT^®^ TKA30 from Performance Chemicals (Belvedere, UK)—potassium acetate dissolved in monoethylene glycol applied as a crosslinking catalyst and characterized by the specific gravity at 25 °C of 0.964. The second catalyst was Dabco 33LV from Air Products (Allentown, USA)—33 wt. % solution of 1,4-Diazabicyclo[2.2.2]octane in dipropylene glycol used as a gelling catalyst. It was characterized by the dynamic viscosity at 25 °C of 125 mPa·s, specific gravity at 25 °C of 1.03, and L_OH_ of 560 mg KOH/g. The last catalyst was dibutyltin dilaurate acquired from Sigma Aldrich (Saint Louis, MO, USA), characterized by specific gravity at 25 °C of 1.066 and tin content of 18.2–18.9%. Distilled water was applied as a chemical blowing agent for prepared PU foams. Ground tire rubber received from Recykl S.A. (Śrem, Poland) was applied as filler for PU-based composites. It was obtained in the process of ambient grinding of post-consumer tires (a mix of passenger cars and truck tires). Applied GTR was characterized by the L_OH_ of 61.7 mg KOH/g.

### 2.2. Modification of Ground Tire Rubber

Thermomechanical treatment of GTR was performed with an EHP 2 × 20 Sline co-rotating twin-screw extruder from Zamak Mercator (Skawina, Poland) as described in our previous work [21]. Briefly, GTR was extruded at the constant feeding rate of 2 kg/h, the barrel temperature was set at 50, 100, 150, or 200 °C, and the screw speed was 50, 100, or 300 rpm. Table 1 presents the impact of thermomechanical treatment on the hydroxyl value (L_OH_) of GTR, determined using the modified test method for isocyanate groups, as described in our other work [21]. The highest hydroxyl values were noted for GTR modified at 100 and 150 °C, pointing to the optimal process temperature, which can accelerate the oxidation occurring during thermomechanical treatment, but on the other hand, may induce partial decomposition of material and reduce the content of functional groups contributing to the hydroxyl value. This effect of excessive GTR oxidation led to the generation of carboxyl groups, which show lower reactivity with isocyanates, hence lower hydroxyl value was noted in our previous work [27]. Considering the impact of screw speed, it shows contradictory effects because increasing this parameter leads to higher shear forces acting on the material, but for a shorter time. Therefore, the overall impact of extrusion parameters on the materials’ properties is not straightforward, which was also reported by other researchers working on extrusion thermomechanical treatment of various materials [28,29,30,31,32].

### 2.3. Preparation of Flexible Polyurethane/Ground Tire Rubber Composite Foams

Composite foams were prepared on a laboratory scale by a single-step method with the isocyanate index of 1:1. A predetermined amount of selected GTR filler (20 parts by weight to the mass of foam) was mixed with the polyols at 1000 rpm for 60 s to guarantee its proper distribution. Afterward, all components were mixed for 10 s at 1800 rpm and poured into a closed aluminum mold with dimensions of 20 × 10 × 4 cm. After demolding, the samples were conditioned at room temperature for 24 h. The amount of reaction mixture poured into the mold was adjusted to obtain foams with a similar level of apparent density, which noticeably affects cellular materials’ performance. As a result, all foams were characterized by an apparent density of 187.5 ± 2.5 kg/m^3^. Table 2 contains the details of foam formulations. Samples were coded according to the type of applied GTR and formulation variant. For example, the sample containing GTR modified at 100 °C, and 50 rpm including the hydroxyl value of GTR in the formulation, was coded as 100/50/OH.

### 2.4. Measurements

After conditioning, foamed polyurethane composites were cut into samples whose properties were later determined following the standard procedures.

The compressive strength of the studied samples was estimated following ISO 604. The cylindric samples with dimensions of 20 × 20 mm (height and diameter) were measured with a slide caliper with an accuracy of 0.1 mm. The compression test was performed on a Zwick/Roell Z020 tensile tester (Ulm, Germany) at a constant speed of 15%/min until reaching 60% deformation.

The tensile strength of foams was estimated following ISO 1798. The beam-shaped samples with 10 × 10 × 100 mm^3^ dimensions were measured with a slide caliper with an accuracy of 0.1 mm. The tensile test was performed on a Zwick/Roell Z020 tensile tester (Ulm, Germany) at a constant speed of 500 mm/min.

Dynamical mechanical analysis (DMA) was performed using a Q800 DMA instrument from TA Instruments (New Castle, DE, USA) at a heating rate of 4 °C/min and the temperature range from −100 to 150 °C. Samples were cylindrical-shaped, with dimensions of 10 × 12 mm.

The thermogravimetric (TGA) analysis of GTR and composites was performed using the TG 209 F3 apparatus from Netzsch (Selb, Germany). Samples of foams weighing approx. 10 mg were placed in a ceramic dish. The study was conducted in an inert gas atmosphere—nitrogen in the range from 30 to 800 °C with a temperature increase rate of 10 °C/min.

## 3. Results and Discussion

**Figure 1:**
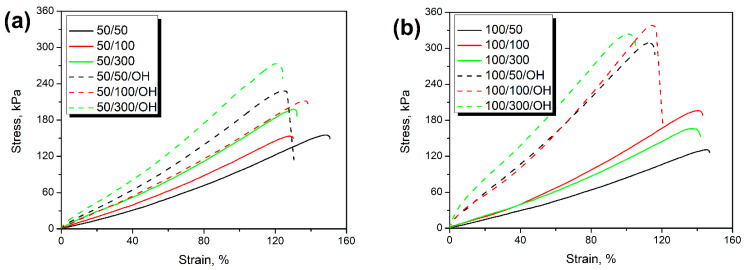

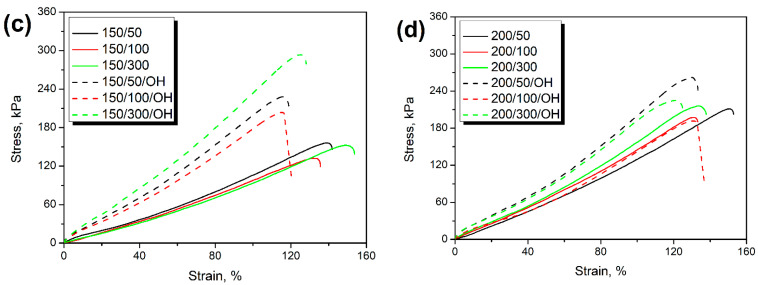
Stress-strain curves of composite foams containing GTR modified at (**a**) 50 °C, (**b**) 100 °C, (**c**) 150 °C, and (**d**) 200 °C.

Figure 1 presents the stress-strain curves for prepared composite foams and provides the results of performed tensile tests. It can be seen that all foams obtained without taking into account the hydroxyl values of GTR were characterized by tensile strength in the range of 134–211 kPa. When the L_OH_ of ground tire rubber was considered during the preparation of foams’ formulations, tensile strength was in the range of 188–339 kPa. Such an effect is associated with the reactions between hydroxyl groups present on the surface of rubber particles and isocyanates. Depending on their extent, hence the hydroxyl value of GTR, these reactions reduce the number of isocyanate groups contributing to polymerization reactions causing weakening of PU structure [27]. When GTR and its hydroxyl values were considered during foams’ preparation, the cumulative amount of isocyanates in the system was higher, resulting in the enhancement of PU phase strength [33]. As presented in Table 1, the lowest L_OH_ values were obtained for GTR particles modified at 200 °C, whose incorporation into PU foams yielded their highest tensile strengths in the range of 204–211 kPa. In the case of these samples, the GTR was less competitive for isocyanates, which did not deteriorate the foams’ performance.

**Figure 2:**
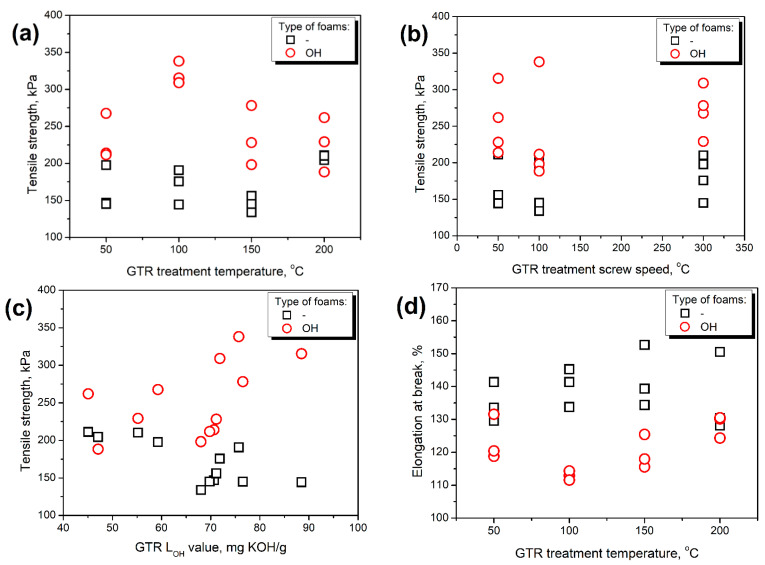

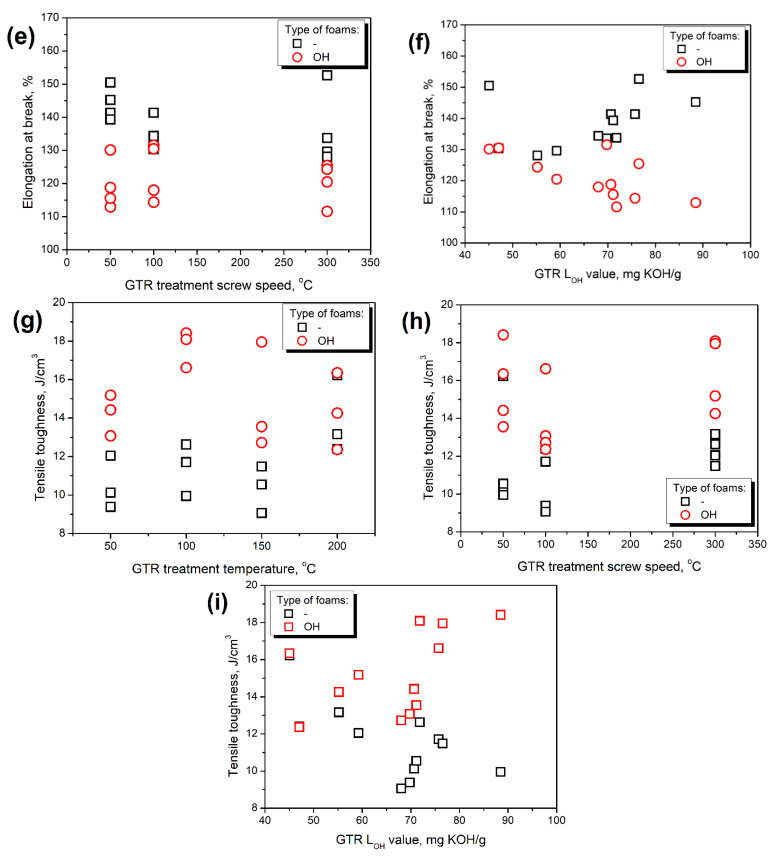
The impact of (**a**,**d**,**g**) GTR treatment temperature, (**b**,**e**,**h**) GTR treatment screw speed, and (**c**,**f**,**i**) GTR LOH value on the (**a**,**b**,**c**) tensile strength, (**d**,**e**,**f**) elongation at break, and (**g**,**h**,**i**) tensile toughness of prepared PU/GTR composite foams.

No direct influence of GTR treatment parameters on their mechanical performance was noted, as presented in Figure 2. The observed pattern was that the tensile strength and tensile toughness, which takes strength into account, showed lower values for the screw speed of 100 rpm compared to 50 and 300 rpm. Such an effect can be explained by the contradictory action of screw speed related to the materials’ residence time in the extruder barrel and the magnitude of shear forces acting on the material [29]. For screw speed of 50 rpm, the material was subjected to a temperature of the extruder barrel and weaker shearing for a longer time, while for 300 rpm, stronger shearing acted on the material for a shorter time. Contradictory effects of screw speed were noted in other works dealing with extrusion-based particle modifications [30,34,35].

The strength of composites, their toughness, and elongation at break more significantly depended on the GTR L_OH_ values resulting from thermomechanical treatment, which is presented in more detail in Figure 2c,f,i. When the hydroxyl value of ground tire rubber was not considered, its increase yielded deterioration of mechanical strength and toughness, which can be attributed to the reduced amount of isocyanate groups taking part in polymerization reactions generating urethane groups. Due to the reduced amount of urethane groups, which act as crosslinks, foams could withstand more extensive deformations, slightly increasing elongation at break [36]. On the other hand, when hydroxyl values of ground tire rubber were considered, more urethane groups were generated. Hence, the PU phase was strengthened, which led to an increase in tensile strength and a drop in elongation at break.

**Figure 3:**
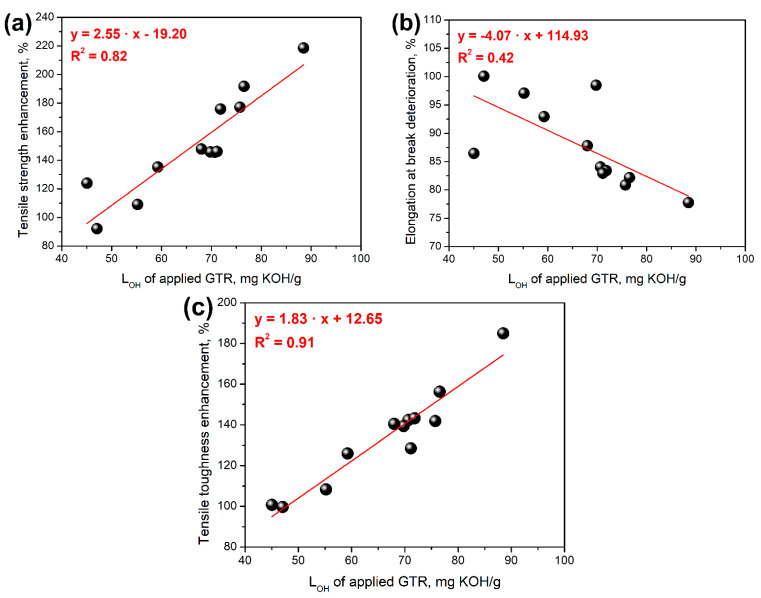
The impact of L_OH_ of applied GTR on the (**a**) tensile strength enhancement, (**b**) elongation at break deterioration, and (**c**) tensile toughness enhancement of PU/GTR composite foams after formulation adjustments.

Figure 2 also shows the evident differences in tensile performance between foams obtained with and without considering the hydroxyl values of GTR. Such an effect is related to the amount of additional isocyanate introduced into the system to compensate for the impact of hydroxyl groups on the surface of GTR particles. For more detailed analysis, Figure 3 shows the relationship between the enhancement of tensile strength and toughness, as well as the drop of elongation at break after formulation adjustments and the L_OH_ values of applied GTR. It can be seen that these relationships are predominantly proportional, which is in line with our previous work dealing with PU/GTR composites prepared with varying isocyanate indexes [22]. Clearly, the presented results confirm literature data suggesting additional crosslinking resulting from the excess of isocyanate groups in the system, which increases tensile strength, enhances toughness, and limits the mobility of polymer macromolecules reducing elongation at break [22,37].

**Figure 4:**
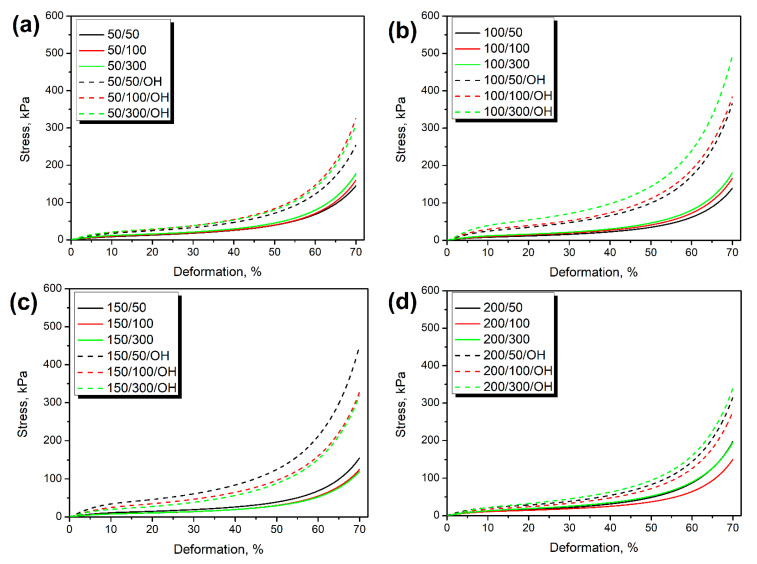
Stress-deformation curves of composite foams containing GTR modified at (**a**) 50 °C, (**b**) 100 °C, (**c**) 150 °C, and (**d**) 200 °C.

**Figure 5:**
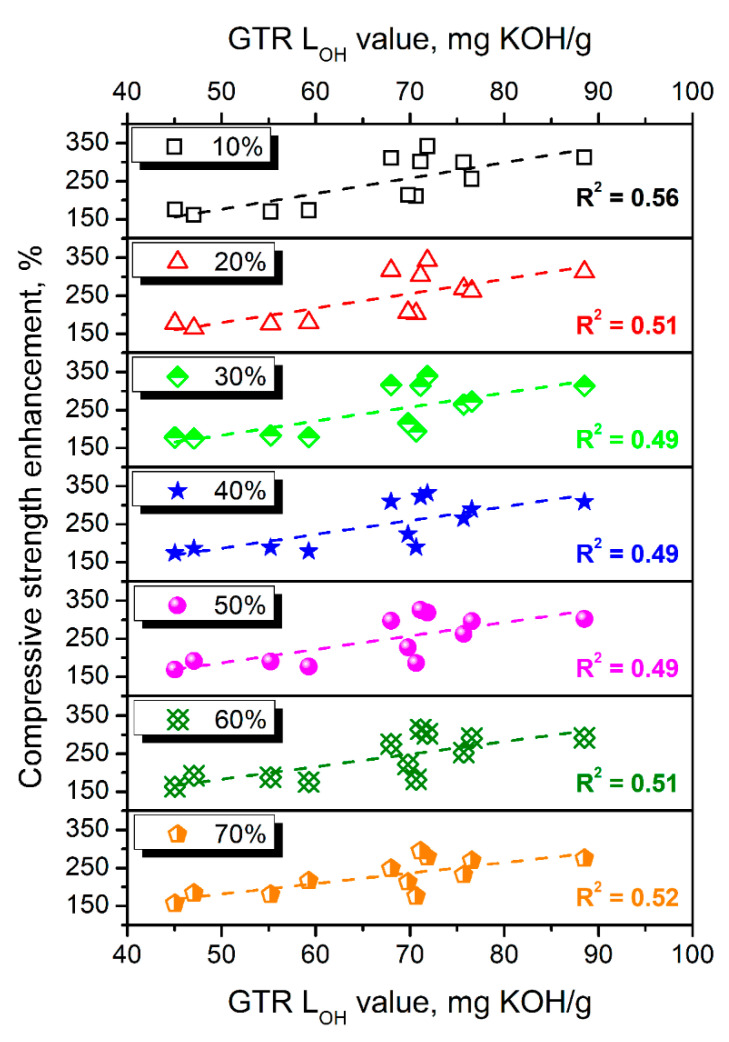
The enhancement of compressive strength at a different level of composites’ deformation resulting from applied formulation adjustments.

Figure 4 shows the static compressive performance of prepared PU/GTR composites and their dependence on the type of introduced GTR particles and applied formulation adjustments. The shape of compressive stress-deformation curves is typical for porous materials, with the gradual strength increase at higher deformation resulting from buckling cell walls and approaching the bulky structure by foams [38]. The significant enhancement of materials’ strength was observed after taking into L_OH_ values of applied rubber particles, which can be presented in Figure 5. It can be seen that the enhancement of composites’ compressive performance was increasing with the GTR hydroxyl value despite some variations from proportionality. Compressive strength values at 50% deformation prior to the significant foams’ densification were increased by 68–224% after formulation adjustments. The strengthening of composites could be attributed to the higher extent of crosslinking resulting from the additional isocyanate reactions [39]. Additional covalent bonds acted as network nodes and reduced the mobility of macromolecular chains inside composites [40]. Therefore, composites require higher external force to reach certain deformation, which the increase in toughness can quantitatively express.

**Figure 6:**
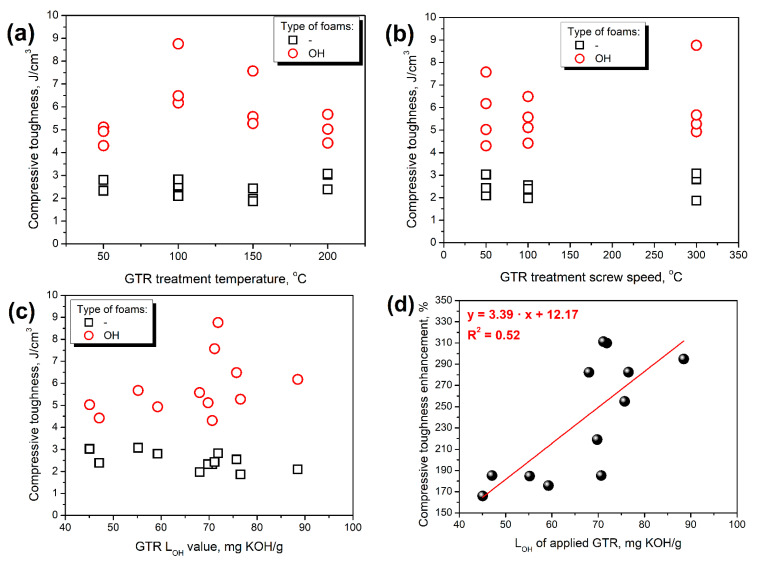
The impact of (**a**) GTR treatment temperature, (**b**) GTR treatment screw speed, and (**c**) GTR L_OH_ value on the compressive toughness of prepared PU/GTR composite foams, and (**d**) the relationship between L_OH_ of applied GTR and compressive toughness enhancement of PU/GTR composite foams after formulation adjustments.

Figure 6 presents the impact of particular parameters of GTR thermomechanical treatment on the value of composites’ compressive toughness, which increased after formulation adjustments along with the compressive strength. Similar to the static tensile tests, the most notable increase in compressive performance, both strength and toughness were noted for composites containing GTR modified at 100 and 150 °C, which can be associated with the highest hydroxyl values (see Table 1). No direct impact of screw speed can be noted. Considering the impact of hydroxyl groups on the surface of GTR particles, the most significant dependence can be observed. Without considering GTR L_OH_ values in formulations, the compressive toughness was lower for composites containing “hydroxyl-richer” GTR particles. Such an effect could be associated with the attraction of isocyanate groups by additional hydroxyls and the weakening of the PU structure [41]. Nevertheless, the performance deterioration was not very significant due to the only partial reactivity of GTR hydroxyl groups with isocyanates, which can be attributed to their lower reactivity compared to polyols, e.g., due to steric hindrance [13]. Compressive toughness increased with GTR hydroxyl values for OH type of foams due to the above-mentioned crosslinking enhancement. What is essential for all discussions related to compressive performance is that all composites were characterized by a similar apparent density, which plays a crucial role in cellular materials [42].

**Figure 7:**
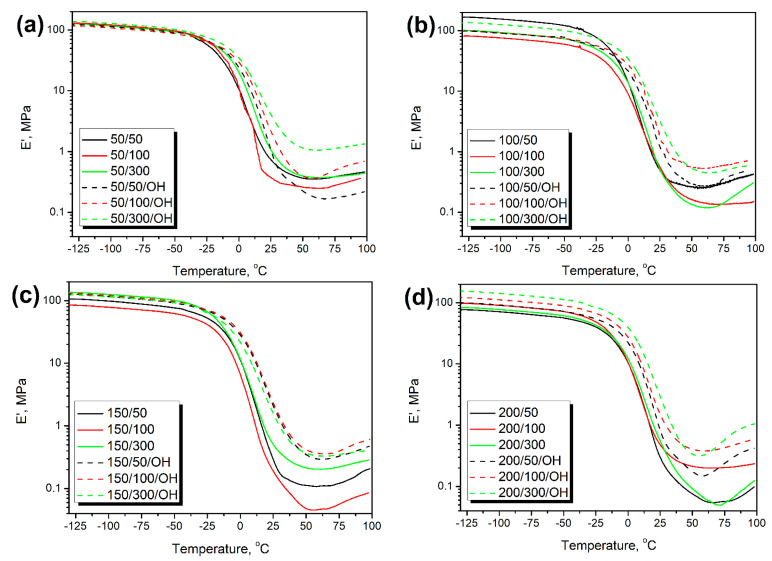
Temperature plots of the storage modulus for PU/GTR foams containing GTR modified at (**a**) 50 °C, (**b**) 100 °C, (**c**) 150 °C, and (**d**) 200 °C.

Figure 7 presents the temperature plots of PU/GTR composites’ storage modulus (E’) obtained during dynamic mechanical analysis. All the analyzed materials show a typical drop of modulus attributed to the glass transition of polymer matrix [43]. The drop of E’ was around two orders of magnitude, as noted in previous works [41,44]. Formulation adjustments resulting from additional hydroxyl groups present on the surface of GTR particles and increased isocyanate content caused stiffening of composites expressed by the E’ rise, which can be seen in the presented graphs. For composites prepared without taking into account the impact of the GTR hydroxyl value, the storage modulus at 25 °C was in the range of 194–1073 kPa, while formulation adjustments shifted the E’ range to 823–4106 kPa. Similar to the enhancement of tensile strength, the stiffening of composites was attributed to the additional crosslinking reactions induced by a higher content of isocyanate groups in the system.

For a more detailed analysis of GTR thermomechanical treatment and formulation adjustments on composites’ stiffness, based on the dynamic mechanical analysis results, the composite performance factor (C factor) was calculated according to the following Equation (1):

C = ((E’_g c_/E’_r c_) / (E’_g m_/E’_r m_)) (1)

where: E’_g_—storage modulus in the glassy state (−100 °C), MPa; E’_r_—storage modulus in the rubbery state (90 °C), MPa; subscripts c and m refer to composite and matrix.

**Figure 8:**
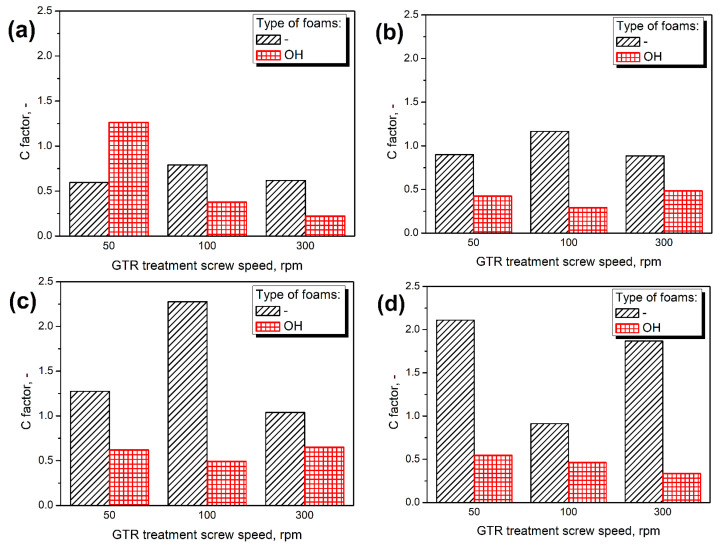
Values of C factor calculated for composites containing GTR modified at (**a**) 50 °C, (**b**) 100 °C, (**c**) 150 °C, and (**d**) 200 °C.

To evaluate the impact of thermomechanical treatment of GTR on the mechanical performance of prepared materials, composite containing unmodified ground tire rubber particles, reported in our previous work [25] reference matrix material. Values of the C factor calculated for prepared composites are presented in Figure 8. They consider the changes in composites’ stiffness resulting from the glass transition of polyurethane matrix. The decreasing C factor indicates higher efficiency of reinforcing effect resulting from the thermomechanical treatment of GTR [45].

Noticeably higher values of C factor were noted for foams prepared without consideration of GTR hydroxyl value, which confirms the results of static mechanical tests. The opposite effect was noted only for composites containing 50/50 GTR particles. The drop in C factor values can be attributed to the partial attraction of isocyanate groups by hydroxyls present on the surface of GTR particles resulting in the strengthening of interfacial adhesion [41]. Moreover, increasing the isocyanate loading to match the total L_OH_ of the polyol mixture, taking into account GTR contribution in most of the samples, led to the C factor values below unity, indicating a reinforcing effect compared to the application of unmodified filler [45].

**Figure 9:**
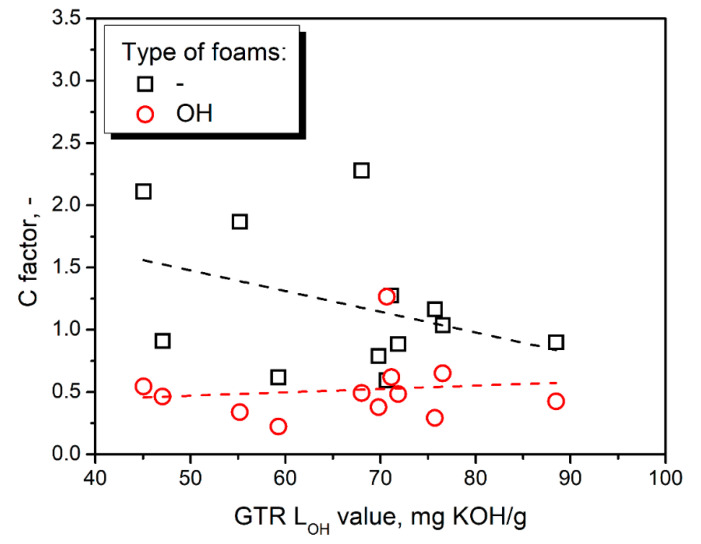
The relationship between GTR L_OH_ values and the C factor of prepared PU/GTR composites.

Figure 9 presents the impact of additional chemical interactions on the composite performance factor. For composites prepared without taking into account the impact of GTR hydroxyl value, the C factor was slightly decreasing with L_OH_, while for the OH-type foams, the trend was hardly noticed. Considering foams without formulation adjustment, increasing hydroxyl values implicate a greater extent of chemical reactions at the interface due to the increasing number of reactive hydroxyls on GTR particles’ surface [46]. However, the impact was not very substantial. When the GTR hydroxyl groups were considered in calculating the required isocyanate amount, the hydroxyl value had a very insignificant impact on the C factor due to the formulation adjustments and tailored isocyanate content in the reacting system.

**Figure 10:**
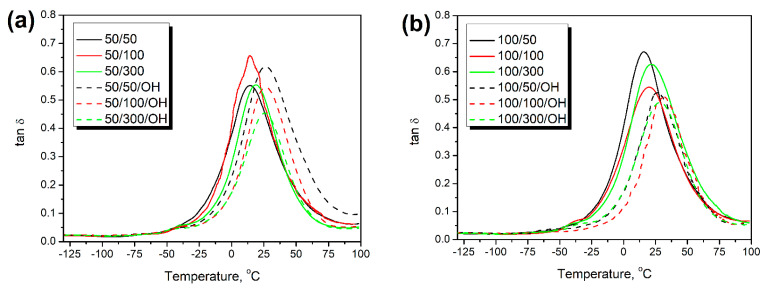

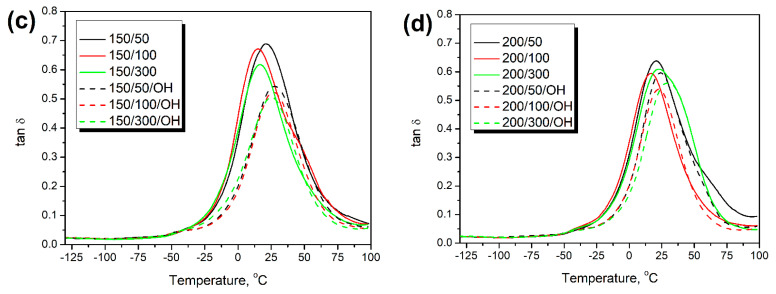
Temperature plots of loss tangent for composites containing GTR modified at (**a**) 50 °C, (**b**) 100 °C, (**c**) 150 °C, and (**d**) 200 °C.

Temperature plots of loss tangent (tan δ) determined during DMA analysis of prepared composite foams are presented in Figure 10. In addition to the information about materials’ stiffness, the dynamic mechanical analysis may provide important insights into their damping behavior. Loss tangent measures a material’s ability to absorb and dissipate mechanical energy [47]. Typically, the incorporation of solid fillers into the polymer matrix enhances the storage modulus at the expense of limiting the loss of tangent peak height [48]. Similar effects were noted in the presented work after formulation adjustments, which can be associated with the limited mobility of polymer macromolecules, confirming the enhanced crosslinking and strengthened interfacial adhesion [49].

**Figure 11:**
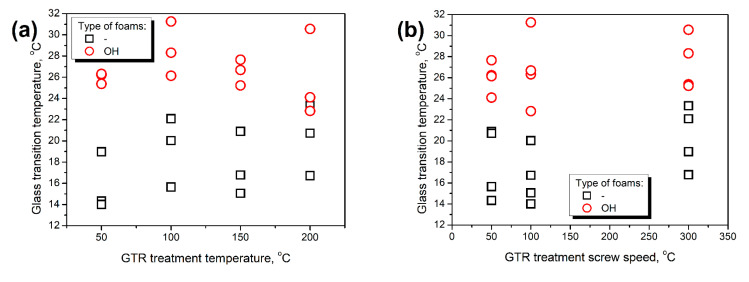

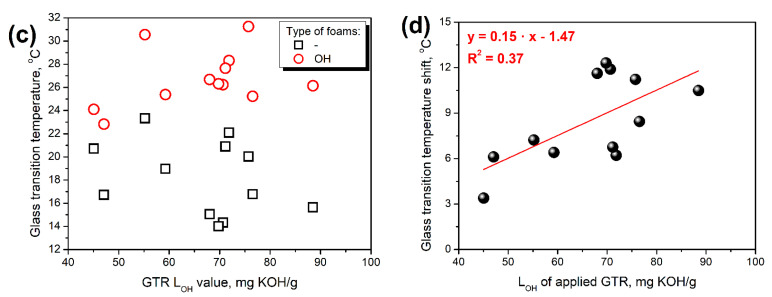
The impact of (**a**) GTR treatment temperature, (**b**) GTR treatment screw speed, and (**c**) GTR L_OH_ value on the glass transition temperature of prepared PU/GTR composite foams, and (**d**) the relationship between L_OH_ of applied GTR and glass transition temperature shift for PU/GTR composite foams after formulation adjustments.

Except for the value of loss tangent, which quantifies the materials’ damping ability, the temperature position of the tan δ peak can be used to determine the glass transition temperature (T_g_). In the case of polyurethane materials, the T_g_ value is susceptible to the balance between isocyanate and hydroxyl groups in the system expressed by the isocyanate index [50,51]. Figure 11 shows that the glass transition temperature was significantly more affected by hydroxyl values of introduced GTR particles than conditions of thermomechanical treatment. Similar to other mechanical properties, foams prepared with and without formulation adjustments can be clearly distinguished by the values of T_g_. Such an effect is attributed to the differences in the above-mentioned balance between isocyanate and hydroxyl groups in the system. This balance was affected by the L_OH_ of GTR and the adjustments of composites’ formulations. Taking the GTR hydroxyl values into account and simultaneously increasing the amount of isocyanate in the system enhanced structural crosslinking by a higher extent of additional reactions leading to allophanates and biurets [12]. As a result, materials require higher energy, so higher temperature, to exceed the Gibbs free energy, increase the free volume and reach the rubbery state. For composites prepared without formulation adjustments, T_g_ was in the range of 14.0–23.3 °C, while after considering GTR hydroxyl values, its values increased to 22.8–31.3 °C. The shift of T_g_ was increasing along with the GTR L_OH_ values. However, the relationship was not perfectly proportional. Other researchers have already reported similar changes in T_g_ induced by increasing the isocyanate index [36,37]. Observed shifts justified the above-mentioned enhancement of composites’ tensile and compressive performance.

**Figure 12:**
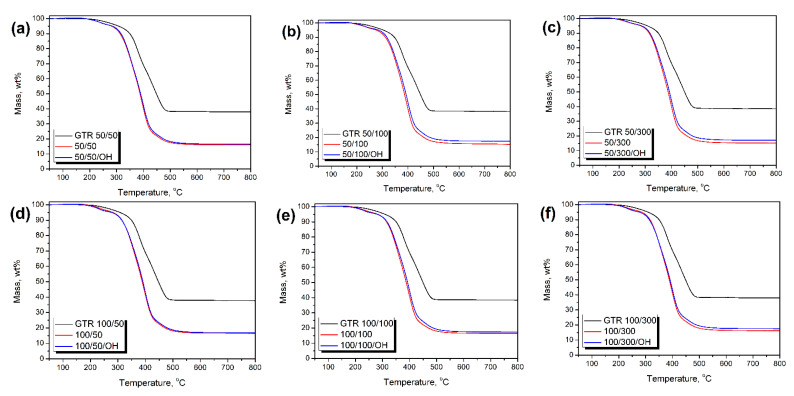

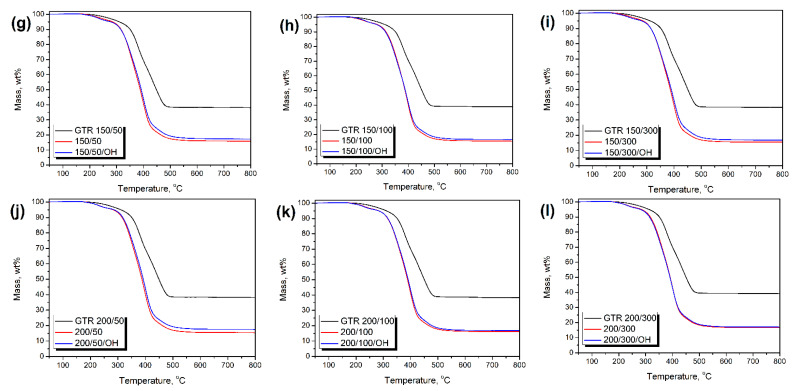
Mass loss curves obtained during thermogravimetric analysis of thermomechanically treated GTR samples and PU composite foams containing different GTR types: (**a**) 50/50, (**b**) 50/100, (**c**) 50/300, (**d**) 100/50, (**e**) 100/100, (**f**) 100/300, (**g**) 150/50, (**h**) 150/100, (**i**) 150/300, (**j**) 200/50, (**k**) 200/100, and (**l**) 200/300.

**Figure 13:**
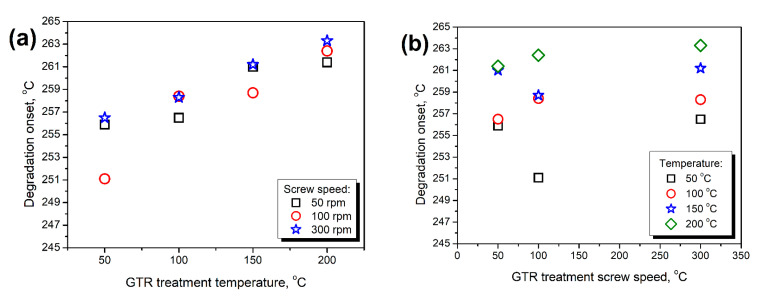

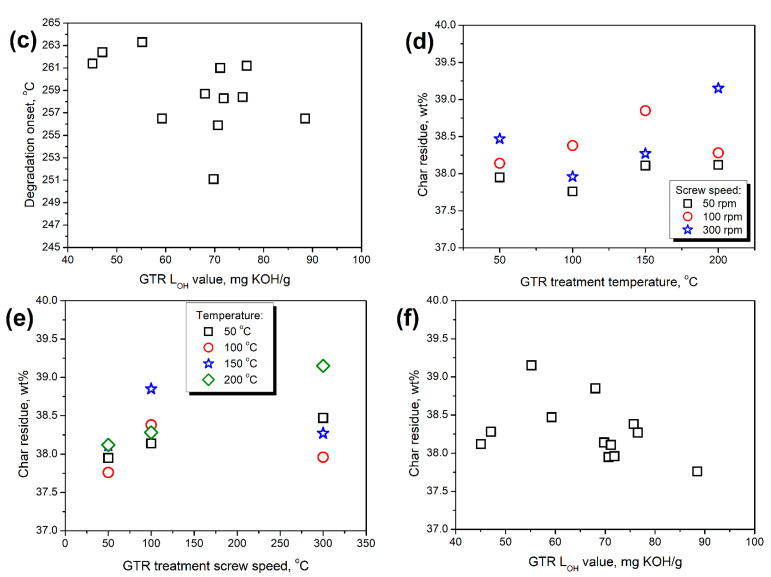
The impact of (**a**,**d**) GTR treatment temperature, (**b**,**e**) GTR treatment screw speed, and (**c**,**f**) GTR L_OH_ value on the (**a**–**c**) degradation onset, and (**d**–**f**) char residue of applied GTR samples.

Figure 12 presents the mass loss curves obtained during thermogravimetric analysis of thermomechanically treated GTR samples and PU composite foams. It can be seen that a similar course of thermal decomposition characterized all applied GTR samples despite different modification conditions. Such an effect can be associated with the chemical composition of GTR, particularly the content of natural rubber and styrene-butadiene rubber [52]. For all the analyzed samples, the onset of thermal degradation, determined as a temperature of 2 wt% mass loss, was in the range of 251.1–263.3 °C. Considering the values of char residue, they were in the range of 37.76–39.15 wt%. Figure 13 provides more insights into the impact of GTR treatment parameters and its hydroxyl value on its thermal degradation parameters. It can be seen that contrary to the mechanical performance, temperature and screw speed applied during thermomechanical treatment showed a more direct impact on the thermal stability of GTR. Hardly any relationship between degradation onset and GTR hydroxyl values can be noted. Such an effect can be related to the partial decomposition of GTR particles’ surface induced by temperature and shear forces acting on material inside the extruder barrel [53].

A similar but not as pronounced impact of thermomechanical treatment conditions was noted on char residue. The actions of temperature and shear forces resulted in the decomposition of lower-molecular weight fractions or partially non-crosslinked portions of material characterized by lower thermal stability during treatment. As a result, the remaining part of the material was characterized by superior thermal stability and higher char residue.

**Figure 14:**
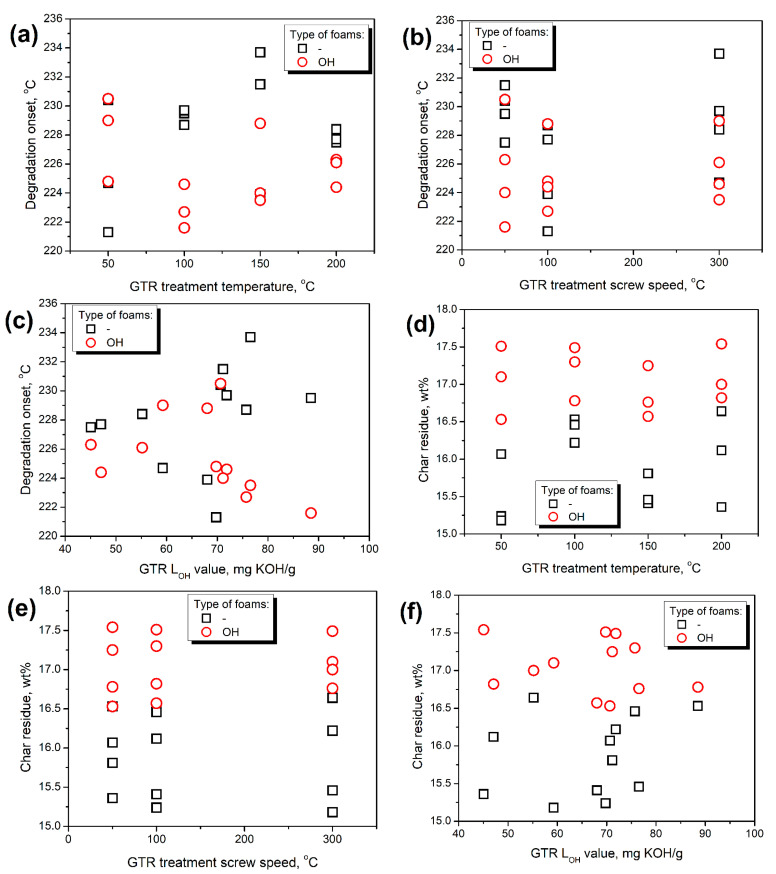
The impact of (**a**,**d**) GTR treatment temperature, (**b**,**e**) GTR treatment screw speed, and (**c**,**f**) GTR L_OH_ value on the (**a**–**c**) degradation onset, and (**d**–**f**) char residue of prepared PU/GTR composites.

Figure 14 presents the impact of GTR treatment parameters and resulting hydroxyl values on the thermal decomposition of prepared composite foams. Despite the noticeable changes in GTR thermal stability induced by varying treatment conditions, the degradation onset of resulting composite foams was hardly affected. Mass loss curves presented in Figure 12 are very similar and for all of the analyzed samples degradation onset was in the range of 221–234 °C, irrespective of the applied GTR type and formulation adjustments. Moreover, no visible trends in thermal stability were noted. Similar to tensile performance, the most noticeable impact was related to the hydroxyl values of GTR. However, the relationships were still far from proportional. Nevertheless, higher hydroxyl values slightly enhanced thermal stability for foams obtained without taking into account the L_OH_ of GTR, while for OH foams, the effect was the opposite. It could be attributed to the formulation adjustments and increased amount of isocyanates in reacting systems leading to the greater extent of reactions yielding urethane, biuret, and allophanate groups, characterized by inferior thermal stability compared to soft segments [54].

Considering char residue, for all prepared composites, its values were between 15 and 18 wt%. No direct impact of GTR treatment conditions or L_OH_ of applied GTR particles was noted. Interestingly, OH foams were characterized by higher values of char residue exceeding 16.5 wt%, which can be associated with higher amounts of isocyanates in the system. Similar findings were reported in previous work [55].

## 4. Conclusions

The presented study was conducted to comprehensively investigate the impact of GTR thermomechanical modification and resulting changes in the L_OH_ values on the stoichiometric balance between isocyanate and hydroxyl groups determining the mechanical, thermomechanical, and thermal performance of foamed PU/GTR composites. The hydroxyl values of applied GTR particles were taken into account during composites’ preparation, whose performance was compared to the samples prepared without formulation adjustments. Obtained results indicated that increasing the content of isocyanates in the system led to the additional stiffening of the PU phase, probably induced by the generation of allophanate and biuret groups. As a result, the molecular motions inside composites’ were affected, which was expressed by a 3.4–12.3 °C shift of the glass transition temperature towards higher temperatures. Such an effect strengthened composites’ structure and increased tensile and compressive strength even by 118 and 243%, respectively. Changes in the composite performance factor calculated from the results of the dynamic mechanical analysis also pointed to the strengthening of materials and the interfacial adhesion between the PU matrix and GTR fillers resulting from formulation adjustments. On the other hand, adjustments reduced composites’ thermal stability, especially for GTR fillers with higher L_OH_ values. Such an effect could be attributed to the inferior thermal stability of hard segments comprised of urethane, allophanate, and biuret groups (all generated more extensively for higher isocyanate contents) compared to soft segments. Reported changes in the composites’ performance resulting from considering the GTR L_OH_ values during formulation development were proportional to the hydroxyl values of GTR, confirming the reactivity of filler functional groups with isocyanates.

Generally, presented results and their dependence on GTR hydroxyl values and applied formulation adjustments pointed to the partial reactivity of GTR functionalities with isocyanates, confirming the importance of undertaken investigations. Moreover, with careful consideration, the presented study could be extended to other PU materials containing reactive additives or fillers, which affect the isocyanate index. Such an approach may provide important insights into developing flexible foamed PU-based composites on an industrial scale.

## Figures and Tables

**Table 1:** Hydroxyl values of thermomechanically modified GTR particles depending on the applied processing conditions.

GTR Treatment Parameters		L_OH_, mg KOH/g	GTR Treatment Parameters		L_OH_, mg KOH/g
Temperature, °C	Screw Speed, rpm		Temperature, °C	Screw Speed, rpm	
50	50	70.65 ± 2.37	150	50	71.13 ± 1.76
	100	69.78 ± 2.43		100	68.00 ± 1.60
	300	59.26 ± 2.81		300	76.55 ± 0.85
100	50	88.49 ± 2.97	200	50	45.05 ± 2.01
	100	75.71 ± 2.92		100	47.08 ± 1.50
	300	71.85 ± 2.01		300	55.21 ± 2.85

**Table 2:** Formulations of PU/GTR composite foams investigated in the presented study.

**Component**	**Type of Applied GTR (Temperature of Treatment/Screw Speed)**											
	**50/50**		**50/100**		**50/300**		**100/50**		**100/100**		**100/300**	
	**-**	**OH**	**-**	**OH**	**-**	**OH**	**-**	**OH**	**-**	**OH**	**-**	**OH**
Rokopol F3000	26.10	26.38	26.10	26.37	26.10	26.27	26.10	26.03	26.10	26.33	26.10	26.13
Rokopol V700	26.10	26.38	26.10	26.37	26.10	26.27	26.10	26.03	26.10	26.33	26.10	26.13
Glycerol	0.60	0.63	0.60	0.63	0.60	0.63	0.60	0.63	0.60	0.63	0.60	0.63
DBTDL	0.50	0.48	0.50	0.48	0.50	0.48	0.50	0.47	0.50	0.48	0.50	0.47
33LV	0.30	0.32	0.30	0.32	0.30	0.32	0.30	0.31	0.30	0.32	0.30	0.32
TKA30	0.30	0.32	0.30	0.32	0.30	0.32	0.30	0.31	0.30	0.32	0.30	0.32
Water	0.30	0.27	0.30	0.27	0.30	0.27	0.30	0.27	0.30	0.27	0.30	0.27
GTR	20.00	16.18	20.00	16.18	20.00	16.90	20.00	16.90	20.00	16.15	20.00	16.90
pMDI	25.80	29.05	25.80	29.06	25.80	28.54	25.80	29.05	25.80	29.18	25.80	28.84
**Component**	**Type of Applied GTR (Temperature of Treatment/Screw Speed)**											
	**150/50**		**150/100**		**150/300**		**200/50**		**200/100**		**200/300**	
	**-**	**OH**	**-**	**OH**	**-**	**OH**	**-**	**OH**	**-**	**OH**	**-**	**OH**
Rokopol F3000	26.10	26.08	26.10	26.41	26.10	26.04	26.10	26.72	26.10	26.71	26.10	26.59
Rokopol V700	26.10	26.08	26.10	26.41	26.10	26.04	26.10	26.72	26.10	26.71	26.10	26.59
Glycerol	0.60	0.63	0.60	0.64	0.60	0.63	0.60	0.64	0.60	0.64	0.60	0.64
DBTDL	0.50	0.47	0.50	0.48	0.50	0.47	0.50	0.48	0.50	0.48	0.50	0.48
33LV	0.30	0.32	0.30	0.32	0.30	0.31	0.30	0.32	0.30	0.32	0.30	0.32
TKA30	0.30	0.32	0.30	0.32	0.30	0.31	0.30	0.32	0.30	0.32	0.30	0.32
Water	0.30	0.27	0.30	0.27	0.30	0.27	0.30	0.27	0.30	0.27	0.30	0.27
GTR	20.00	16.90	20.00	16.20	20.00	16.90	20.00	16.39	20.00	16.39	20.00	16.32
pMDI	25.80	28.94	25.80	28.97	25.80	29.04	25.80	28.12	25.80	28.14	25.80	28.47

## Data Availability

The data presented in this study are available in Insights into stoichiometry adjustments governing the performance of flexible foamed polyurethane/ground tire rubber composites.
